# Effect of domain knowledge encoding in CNN model architecture—a prostate cancer study using mpMRI images

**DOI:** 10.7717/peerj.11006

**Published:** 2021-03-09

**Authors:** Piotr Sobecki, Rafał Jóźwiak, Katarzyna Sklinda, Artur Przelaskowski

**Affiliations:** 1 Applied Artificial Intelligence Laboratory, National Information Processing Institute, Warsaw, Mazowieckie, Poland; 2 Faculty of Mathematics and Information Science, Warsaw University of Technology, Warsaw, Poland; 3 Department of Radiology, Centre of Postgraduate Medical Education, Warsaw, Poland

**Keywords:** Artificial intelligence, Machine learning, Prostate cancer, PI-RADS, mpMRI, Prostate cancer diagnostics, Knowledge-based modeling, Neural network architectures, Deep learning, Multimodal convolutional neural networks

## Abstract

**Background:**

Prostate cancer is one of the most common cancers worldwide. Currently, convolution neural networks (CNNs) are achieving remarkable success in various computer vision tasks, and in medical imaging research. Various CNN architectures and methodologies have been applied in the field of prostate cancer diagnosis. In this work, we evaluate the impact of the adaptation of a state-of-the-art CNN architecture on domain knowledge related to problems in the diagnosis of prostate cancer. The architecture of the final CNN model was optimised on the basis of the Prostate Imaging Reporting and Data System (PI-RADS) standard, which is currently the best available indicator in the acquisition, interpretation, and reporting of prostate multi-parametric magnetic resonance imaging (mpMRI) examinations.

**Methods:**

A dataset containing 330 suspicious findings identified using mpMRI was used. Two CNN models were subjected to comparative analysis. Both implement the concept of decision-level fusion for mpMRI data, providing a separate network for each multi-parametric series. The first model implements a simple fusion of multi-parametric features to formulate the final decision. The architecture of the second model reflects the diagnostic pathway of PI-RADS methodology, using information about a lesion’s primary anatomic location within the prostate gland. Both networks were experimentally tuned to successfully classify prostate cancer changes.

**Results:**

The optimised knowledge-encoded model achieved slightly better classification results compared with the traditional model architecture (AUC = 0.84 vs. AUC = 0.82). We found the proposed model to achieve convergence significantly faster.

**Conclusions:**

The final knowledge-encoded CNN model provided more stable learning performance and faster convergence to optimal diagnostic accuracy. The results fail to demonstrate that PI-RADS-based modelling of CNN architecture can significantly improve performance of prostate cancer recognition using mpMRI.

## Introduction

In 2018, it was estimated that prostate cancer (PCa) was the second most common type of cancer globally, contributing to 3.8% of all deaths from the disease (Rawla, 2019). It is estimated that one in seven males will suffer from PCa during their lifetime. The detection and characterisation of clinically significant prostate cancer (csPCa) within the prostate gland is a complex process. A significant breakthrough came with the emergence of multi-parametric magnetic resonance imaging (mpMRI), which utilises a combination of anatomical and functional pulse sequences, and has quickly become a cornerstone in the diagnostic algorithm of csPCa (Polanec et al., 2020). Current mpMRI methods include conventional T2-weighted imaging (T2W), diffusion-weighted imaging (DWI) with apparent diffusion coefficient (ADC) mapping, and dynamic contrast-enhanced MRI (DCE). mpMRI has proved to be an effective technique for localising high-risk prostate cancer, in addition to guiding biopsies, and better reflects the true Gleason grade (Fei, 2017). As a result, recent guidelines issued by the European Association of Urology strongly recommend that patients are referred for mpMRI prior to biopsies (Mottet et al., 2017). However, the introduction of mpMRI to clinical practice has also brought new challenges. Prostate mpMRI imaging depends heavily on the vendors of MRI equipment, and the parameters used—including magnet field, gradient strength, and choice of sequence parameters. Moreover, the prostate mpMRI interpretation process is characterised by high inter-observer variability, and the learning curve effect.

### Prostate Imaging-Reporting and Data System

The Prostate Imaging Reporting and Data System (PI-RADS) was introduced by the European Society of Urogenital Radiology (ESUR) in 2012, with the aim of standardising prostate mpMRI examination protocols and suspicious lesion reporting. The PI-RADS system categorises prostate lesions based on the likelihood of cancer according to a five-point scale. The current version, PI-RADS 2.1, was launched in 2019. Its clinical utility is growing, and several studies have confirmed that PI-RADS scoring improves the diagnostic accuracy of mpMRI (Hamm & Asbach, 2018).

### Deep learning and domain knowledge encoding

The recent success of deep learning methodology exploits the concept of end-to-end models learned directly from data (data-driven modelling). This stands in opposition to the past dominance of hand-crafted feature engineering (knowledge-based modelling), in which domain knowledge usually plays a central role, and the majority of the architecture is manually hard-wired, based on domain expertise (Muralidhar et al., 2018). The question remains unresolved of how much domain knowledge is necessary for learning in domain-agnostic situations in which no prior knowledge is assumed, but is rather induced from the data. Another question arising is how prior knowledge can be encoded within deep neural networks. In the case of deep learning, the process of domain knowledge incorporation might relate to the selection of general network architecture. Different classes of neural network are preferred, depending on the nature of the data being processed, and the aim of the task being undertaken. For example, recurrent neural networks are frequently advocated for data with sequential structures (Muralidhar et al., 2018). At this level of generality, handling images and video processing tasks usually involves the selection of convolution neural networks (CNNs). Convolution is a powerful concept for constructing a robust feature space based on image data.

An alternative approach assumes integration of the knowledge-based theoretical approach to data-driven empirical modelling (Todorovski & Dzeroski, 2006). Such a conception of knowledge-based modelling formulated in linguistic terms, with ontological structures or any other representations embedded in reasoning procedures, aims to improve knowledge or understanding of a phenomenon. The concept of hybrid modelling has already been employed in the field of biomedical informatics (Pivovarov & Elhadad, 2012). The first attempts in the field of neural networks aimed at modifying network architecture to reflect current domain knowledge were influenced by earlier ideas implemented in relation to the structures of classic neural networks (Davies, 1991; DeClaris & Su, 1993). Recently, integration of prior knowledge into deep learning has been enthusiastically developed, and an interesting trend is becoming apparent in the development of deep learning models (Diligenti, Roychowdhury & Gori, 2017; Futia & Vetro, 2020).

Lately, research has addressed the problem of deep neural network instability due to perturbations in visual input, resulting from image processing procedures (such as compression and cropping), or diversified sources of training data (Zheng et al., 2016; Strisciuglio, Lopez-Antequera & Petkov, 2020). Data augmentation is frequently insufficient. Moreover, this form of robustness must be learned from augmented input data, and is only specific for classes of perturbation which are effectively represented by that data. The same lack of robustness can be observed in the case of biomedical images (Tasdizen et al., 2018), in which additional data diversification occurs due to different acquisition protocols and machine vendors, or as a result of image multi-modality, which increases the complexity of learning. In Kloenne et al. (2020) the introduction of domain-specific data pre-processing and augmentation to state-of-the-art CNN architectures improved the network’s robustness, and stabilised the prediction performance on a range of tasks, such as liver and kidney segmentation.

### Deep learning in prostate cancer—related work

CAD systems used in mpMRI-based examinations play the role of a second observer, providing a method of reporting the probability of a finding being clinically significant in an unbiased manner. Presently, deep learning models are establishing a new state of the art in the field of medical data analysis, and specifically in the area of prostate cancer diagnosis with mpMRI.

The problem of csPCa detection can be formulated in two different ways: as a classification problem (Song et al., 2018; Wang et al., 2017; Yang et al., 2017a; Le et al., 2017; Yang et al., 2017b); or as a semantic-segmentation problem (Ishioka et al., 2018; Alkadi et al., 2019; Kiraly et al., 2017; Schelb et al., 2019). In the first case, a patch-based classification of suspected tissue samples is typically performed, which retrospectively exploits annotated image patches. The second approach utilises a pixel-level classification; the goal of which is to assign a label to each pixel, indicating its association to a proper class (usually cancer tissue, normal organ, or background). The selection of basic architecture depends heavily on csPCa task formulation. For the classification approach, VGG (Song et al., 2018; Le et al., 2017), ImageNet (Wang et al., 2017), GoogLeNet (Yang et al., 2017a; Le et al., 2017; Yang et al., 2017b), and ResNet (Le et al., 2017) have been used. Encoder–decoder architectures are usually preferred in the semantic-segmentation approach, promoting models such as U-Net (Ishioka et al., 2018; Schelb et al., 2019), ResNet (Ishioka et al., 2018), SegNet (Kiraly et al., 2017), and VGG16 (Alkadi et al., 2019).

One crucial aspect which occurs widely in medical imaging is the multi-modality of image data. In the case of prostate cancer, data multi-modality is expressed in the multi-parametric form of MRI scans. The problem of multi-modality fusion in CNNs was analysed extensively by Zhou, Ruan & Canu (2019), in which the authors proposed various multi-modal fusion strategies. Most of the solutions in the area of prostate cancer detection exploit the concept of input-level fusion or that of decision-level fusion. In the input-level fusion strategy, multi-parametric images are fused before being passed to the network. The most common form of input-level fusion is image registration, in which co-registered multi-parametric image series constitute an input for network training (Kiraly et al., 2017; Song et al., 2018). The conception of decision-level fusion usually assumes the use of individual networks for each multi-parametric series (Yang et al., 2017a; Le et al., 2017; Yang et al., 2017b; Schelb et al., 2019). Each network can learn unique and mutually complementary information from different multi-parametric images. This allows the creation of modality-specific feature representations. The results from individual networks are integrated and fused at the classification stage, and reach a final decision.

In this work, we hypothesise that encoding prior domain knowledge to state-of-the-art CNN architecture in the task of csPCa detection on mpMRI images can improve the robustness of a CNN model, and stabilise its learning. We assume that the optimised architecture of the CNN, which reflects prior knowledge of the diagnostic process encoded in the PI-RADS rules, can provide an inductive bias, which allows to prioritise interpretation of diagnostic information according to a lesion’s location in the prostate zone.

## Materials and Methods

### PI-RADS as a source of domain knowledge

PI-RADS v2 introduced the concept of a dominant mpMRI sequence, related to the original location of a lesion. Peripheral zone (PZ) lesion assessment is based primarily on DWI evaluation, with DCE playing a supporting role in cases in which the evaluation is inconclusive. Similarly, for the transition zone (TZ), the T2W evaluation is primary, and DWI plays a supporting role. Consequently, the assignment of an overall score to a lesion, indicating the likelihood of clinically significant prostate cancer, is based on scoring related to the dominant sequence, with the possibility of minor modification, based on the assessment score of other sequences. Furthermore, the interpretation of DCE is simplified only to include ‘positive’ or ‘negative’ (Becker et al., 2017). Reporting of lesions located in other zones, such as in the central zone (CZ), anterior fibromuscular stroma (AFS), or seminal vesicles (SV) is usually performed according to the rules applying to the nearest neighbouring zone, or to the zone from which the lesion appears most likely to have originated. For the purpose of this study, based on interviews with radiology specialists, evaluation of lesion in SV and AFS is performed as those in TZ are.

PI-RADS assessment scoring rules for individual mpMRI sequences are based on groups of significant imaging features, such as those related to signal intensity, lesion margin, and shape. The criterion for the presence of cancer in T2W is a low-signal intensity mass or nodule located in the PZ, which is hypointense compared to normal tissue, and has ill-defined margins (Aydın, Kizilgoz & Tekin, 2015). However, TZ lesions that appear as focal hypointense areas may mimic PCa. For DWI, the key diagnostic criterion in the detection of prostate cancer is the focal or conglomerated areas, which are hyperintense in both DWI and ADC mapping, relative to the surrounding prostate tissue. In the case of DCE, the presence of PCa is related to asymmetric high-contrast enhancement, particularly early nodular enhancement (Aydın, Kizilgoz & Tekin, 2015). Determining the individual nature of suspicious lesions on each of the mpMRI sequences and establishing their mutual correlation have proved crucial for the effectiveness of PCa diagnosis. They may also play an important role in developing machine learning algorithms dedicated to the recognition of prostate cancer.

### Data

In this study, a publicly available database of mpMRI data for prostate lesion classification was used, which was originally created for the PROSTATEx Challenge (SPIE-AAPM-NCI Prostate MR classification Challenge) held in conjunction with the 2017 SPIE Medical Imaging Symposium (Litjens et al., 2017). The database incorporates the data of 344 patients, divided into a training set (204 patients with 330 suspicious findings), and a test set (140 patients with 208 suspicious findings). Suspicious findings in the dataset were annotated with their locations, prostate zones, and clinical significance. The findings were located in four separate prostate zones. The dataset was imbalanced, as there were more insignificant lesions (254; PZ 155 / TZ 73 / AFS 24 / SV 2) than significant ones (76; PZ 36 / TZ 9 / AFS 31 / SV 0). The clinical significance of each finding on mpMRI was set on the basis of the initial PI-RADS assessment, which qualified lesions for further biopsy verification. Findings with a PI-RADS score of 2 or lower were not biopsied, and marked as clinically insignificant. Other findings (PI-RADS > 2) were biopsied and assessed using the Gleason Scoring (GS) system, which offers both prognostic and risk data stratification (Blute et al., 2001). Findings with a GS score of 7 or above were marked as clinically significant.

### Normalization, VOI selection and data augmentation

In order to compensate for the varying parameterisation of medical image acquisition methods and inter-patient variability, all images were first normalised and min-max scaled. A form of median normalisation, originally proposed by Kwak et al. (2015) was utilised, preceded by the identification and removal of potential outliers. Other normalisation methods were considered, as proposed in previous research (Sobecki et al., 2017). Median normalisation, however, achieved the best model performance.

After the normalisation step, the volumes-of-interest (VOIs) surrounding each lesion were extracted. According to PI-RADS v2.1 standard, lesions greater than 1.5 cm in size are to be reported as findings of high probability of clinical significance (the highest PI-RADS score). We decided to extract 3 cm × 3 cm × 3 cm VOIs located in the centre of the lesions. Extracting lesions and the surrounding regions offers important contextual information. The Extracted VOIs were not interpolated; therefore, the volume dimensions from different multi-parametric image series varied due to the varying voxel spacing between the mpMRI sequences.

In order to increase the size of the training set, we used both online and offline data augmentation. Prior to model optimisation (offline augmentation), each VOI was randomly rotated ten times. The rotation degree was selected randomly within the range, (−90, +90). The rotated VOIs were stored locally. This allowed the training dataset to be augmented to 3 300 cases. During training, we used the following augmentation methods (online augmentation in the training pipeline) applied with random probabilities and parameterisation: brightness and contrast modification, Gaussian noise addition and volume flipping. The variables used for parametrisation of those methods were normal random variables with distributions: *N*(1.0, 0.5625) for contrast factor (*σ* = 0.75), *N*(0, 0.01) for brightness shift (*σ* = 0.1), and *N*(0, 0.001) for white noise addition (*σ* = 0.01). The variances of those distributions were selected experimentally. Additionally small, random translations (up to: ±12 voxels in plane for T2W, ±4 for DCE, ±3 for DWI; ±2 slices for T2W, DWI and DCE) were performed. The approximate distance between slices is three mm for all T2W, DWI and ADC imaging. The size of single voxel is related to approximate resolution of each mpMRI imaging modality: 1.5 × 1.5 × 3 mm for DCE, 2 × 2 × 3 mm for DWI and 0.5 × 0.5 × 3 mm for T2W. Thus, the translations made were within the range of ±6 mm in all dimensions.

### CNN models

**Figure 1:**
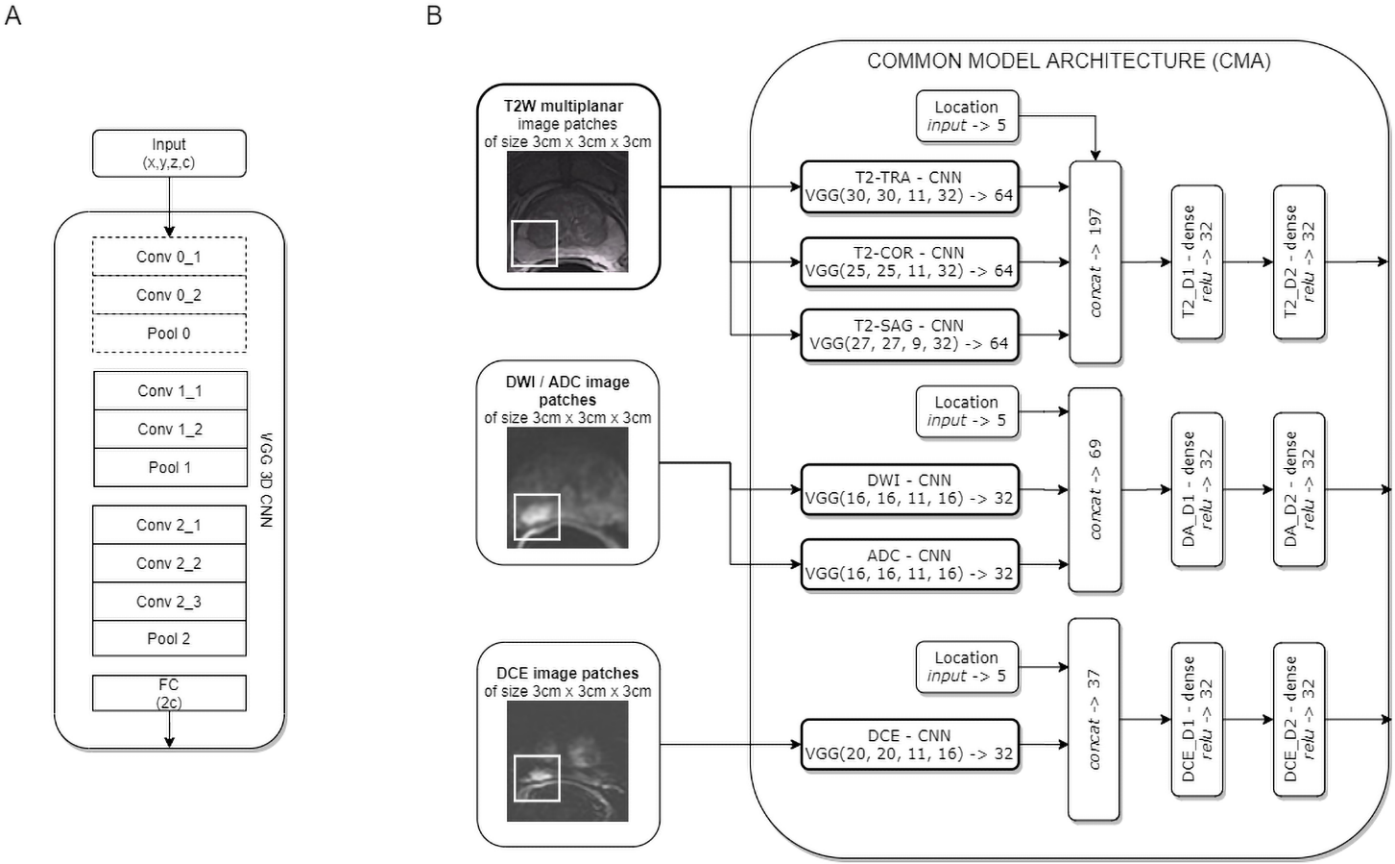
Common model architecture (CMA) implementing the data-driven selection of modality-specific features from functional and anatomical forms of mpMRI (B); the CMA implements the parallel processing of multi-modal mpMRI data in sub-networks, which are variants of the VGG 3D model (A). The convolution layers marked with a dashed line are used only for T2W images.

Two CNN models for clinically significant prostate cancer recognition were subjected to comparative analysis. Due to the characteristics of the PROSTATEx dataset, in which lesion centre coordinates are defined, a patch-based approach for csPCa detection was proposed. Both CNN models share a common component of the architecture, which is presented in Fig. 1.

The common model architecture (CMA) uses individual networks for each multi-parametric image sequence to calculate modality-specific feature representations. Each mpMRI image series (T2W, DWI, DCE) is processed using a variant of the VGG network. The proposed model builds upon the VGG-16 core network (Simonyan & Zisserman, 2014) to a 3D model by introducing 3D convolutional layers instead of 2D ones. One advantage of the VGG 3D model is the use of small 3 × 3 × 1 kernels that allow the architecture to adapt to diversified input volume dimensions. The proposed VGG 3D architecture is presented in Fig. 1A, while the detailed parameterisation is presented in Table 1. Dropout (with 0.125 probability) and L2 normalisation were applied on hidden dense layers. In the case of T2W modality, images from the sagittal, coronal, and transverse planes were processed independently in individual VGG 3D networks. Moreover, a single VGG 3D consists of an additional convolutional-pooling block (marked with a dotted line in Fig. 1A), due to the higher resolution of T2W modality, in which images are acquired with smaller voxel spacing (0.5 × 0.5 × 3 mm). DWI and ADC images are processed in separate networks. Similarly, the DCE sequences are processed independently. In practice, we used K^Trans^ sequences, which allowed us to analyse the quantitative parameters of the DCE MRI time series. Feature vectors from the individual VGG 3D CNN are concatenated at the level of individual modalities, with additional information about lesion locations binary encoded in the form of a five-element vector, with elements corresponding to the prostate zones. After concatenation, modality-specific features are passed to dense layers. Finally, the CMA produces three modality-specific 32-element feature vectors.

**Table 1:** Parameterised VGG-inspired modality CNN architecture—where x and y correspond to layer width and height, z to the layer depth, and c to the number of channels. Conv 0_1, Conv 0_2, and Pool 0 are additional layers used only for T2W modality. The first pooling layers have a depth stride of 1, while the last ones have a depth stride of 2, owing to the DICOM data dimensionality with a different resolution in the X, Y, and Z axes.

Id	Operation	Filter	Strides	Width	Height	Depth	Channels
Conv 0_1	Convolution	3 × 3 × 1	1 × 1 × 1	2x	2y	z	*c*∕2
Conv 0_2	Convolution	3 × 3 × 1	1 × 1 × 1	2x	2y	z	*c*∕2
Pool 0	Max pooling	3 × 3 × 1	2 × 2 × 1	x	y	z	*c*∕2
Conv 1_1	Convolution	3 × 3 × 1	1 × 1 × 1	x	y	z	c
Conv 1_2	Convolution	3 × 3 × 1	1 × 1 × 1	x	y	z	c
Pool 1	Max pooling	3 × 3 × 1	2 × 2 × 1	*x*∕2	*y*∕2	z	c
Conv 2_1	Convolution	3 × 3 × 3	1 × 1 × 1	*x*∕2	*y*∕2	z	2c
Conv 2_2	Convolution	3 × 3 × 3	1 × 1 × 1	*x*∕2	*y*∕2	z	2c
Conv 2_3	Convolution	3 × 3 × 3	1 × 1 × 1	*x*∕2	*y*∕2	Z	2c
Pool 2	Max pooling	3 × 3 × 3	2 × 2 × 2	*x*∕2	*y*∕2	⌊*z*∕2⌋ + 1	2c
FC	Average pooling	global	global	–	–	–	2c

**Figure 2:**
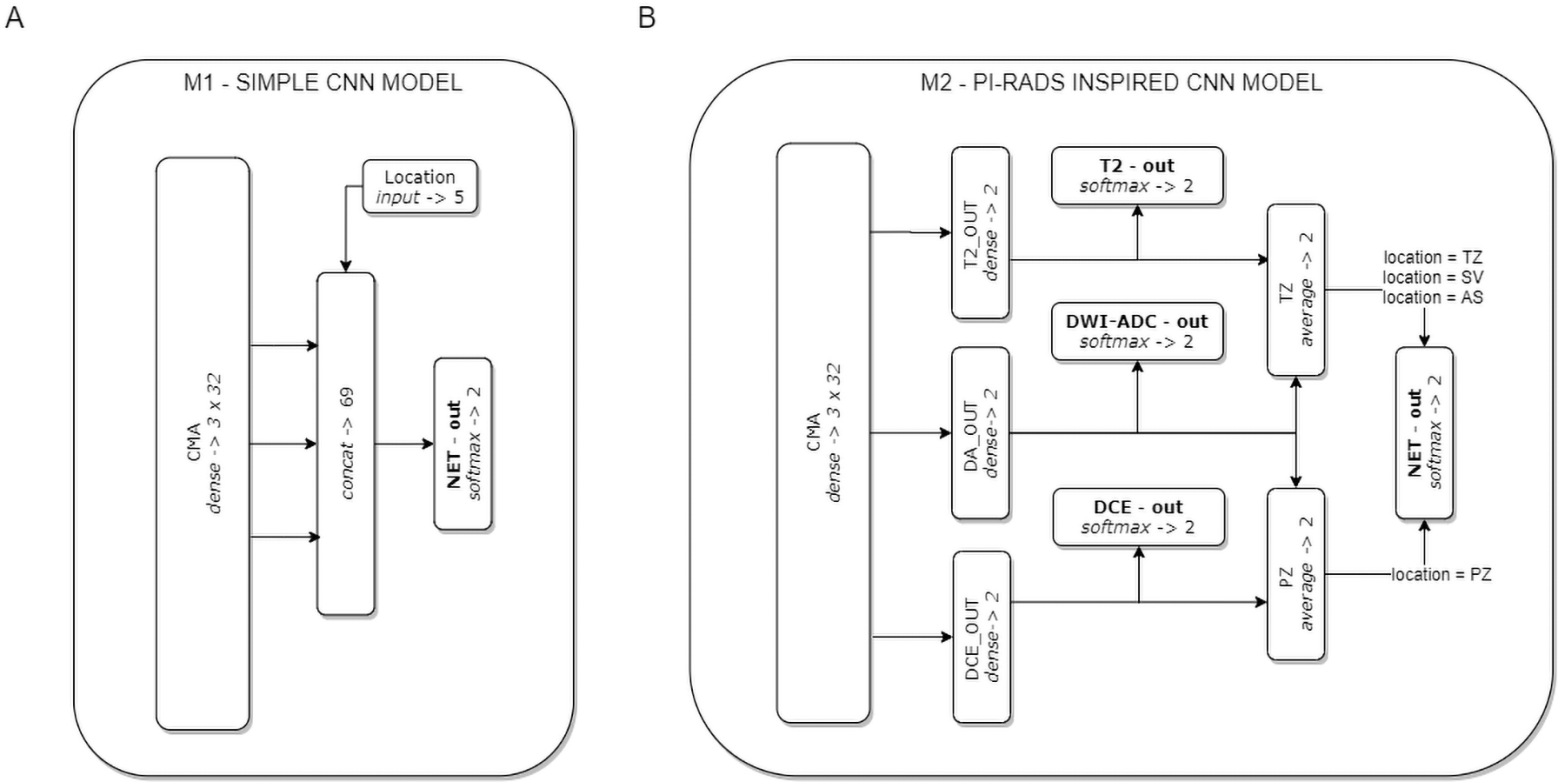
The two CNN models used in the experiments. Model M1 (A) implements decision-level fusion of complementary information from different mpMRI modalities. The output of model M2 (B) is knowledge-based optimised according to the PI-RADS decision rules (the averaged classification results from selected modality-specific sub-networks related to lesion location in the PZ or TZ zones).

On the basis of the CMA, two CNN models were formulated (see Fig. 2). Model M1 represents a CNN architecture with simple decision-level fusion of complementary information from different modalities. Three modality-specific feature vectors from the CMA are directly concatenated and passed to a dense softmax layer, which implements the classification output.

Model M2 implements the concept of domain knowledge encoding in a model architecture, inspired by PI-RADS assessment, in which the final decision on lesion malignancy depends on the location of a lesion within the prostate gland, and its features assessed on location-related dominant sequences. In model M2, three modality-specific feature vectors from the CMA constitute sub-networks related to individual mpMRI modalities (T2, DWI-ADC, and DCE). Each sub-network is provided with an auxiliary classifier appended behind each modality-specific feature extractor, to keep the independently learned features separated. The utilisation of auxiliary classifiers stems from the Inception DCNN network architectures (Szegedy et al., 2015). During training, the losses from these auxiliary classifiers were added to the main classification loss. At the point of inference, these auxiliary classifiers are discarded. The final M2 model decision is based on the location of the lesion within the prostate gland. Two stream raw predictions (PZ and TZ) are created based on the optimised modality-specific sub-networks. The outputs from the two selected sub-networks are included and averaged (DA-out and DCE-out, or T2-out and DA-out for PZ and TZ, respectively) to formulate the final logits for each stream. The final probability of lesion malignancy is produced on an output of the soft-max layer (NET-out), to which logits are routed from a suitable stream. Logits routing to the output of model M2 express the rules of the PI-RADS decision-making process.

### Experiments

Both models were trained on the same training set. Complex loss function, used for the optimisation of model M2, is composed of the loss values of model sub-networks (Eq. (1)). In effect, the top-level network, capable of generating predictions based on all modalities is trained simultaneously with sub-networks basing their predictions on single modalities. The relatively small dataset and complex model architecture required measures to combat model overfitting. L2 regularisation loss was introduced at dense layers, and added to the total loss of the model:

(1) \begin{eqnarray*}l(x)= \frac{\sum _{i=1}^{{n}_{i}}{l}_{i}\ast {w}_{i}}{\sum _{i=1}^{{n}_{i}}{w}_{i}} +0.1 \frac{{l}_{L2}}{{n}_{L2}} \end{eqnarray*}

Effectively, the total loss is the sum of the weighted average of sub-losses, where: *n*_*l*_—number of minor losses, *l*_*i*_ –minor loss value, *w*_*i*_—weight of each minor loss, *l*_*L*2_—L2 regularisation loss, and *n*_*L*2_—total number of L2 regulated layers. To calculate the total loss, we evaluated the weighted average of cross-entropy mini-batch values to obtain the total cost of the model; the weights depended on the network layer output and lesion location (Table 2). Those values were set experimentally, and have not been normalised for ease of fine-tuning.

Model optimisation with mini-batch stochastic gradient descent (with a momentum value of 0.9) was performed for a maximum number of 500 epochs. Additionally, the experiments were repeated, learning the model for 25, 50, 75 and 100 epochs respectively. Other hyperparameters, as shown in Table 3, were tuned empirically to achieve optimal model generalisation capabilities, and the best accuracy. The selected optimal hyperparameters were set to the same values between the models analysed to avoid unnecessary freedom in methodology.

Five-fold cross-validation (CV) was used for the evaluation of each CNN model. The models were built on 80% of the training data, and the remaining 20% was held out for each model validation. The whole learning experiment was repeated twice, resulting in ten fully optimised versions of both models (*n* = 10). For each training iteration, selected learning samples were shuffled and queued by ten CPU threads responsible for online data augmentation, while model optimisation was performed on the GPU. The optimal model identification was related to the best obtained AUC score on the validation subset for all CV iterations (the validation samples were not online augmented). The prediction used to evaluate the test set was the mean probability prediction of the ten best-performing models from the training phase.

**Table 2:** Minor loss weights (w) for CNN sub-networks and lesion locations. The loss for model M1 includes only the output of the whole network (NET-out). For model M2, sub-network auxiliary losses are also included. M2 loss varies for lesions located in the TZ and PZ zones, reflecting domain knowledge resulting from the PI-RADS rules. A small weight value is included for complementary setup in order to enforce the use of the data for training, as well as for comparison purposes.

i	subnet	zone	M1	M2
0	NET	–	100	100
1	DCE	TZ	–	5
2	DCE	PZ	–	20
3	T2	PZ	–	5
4	T2	TZ	–	20
5	DWI_ADC	PZ	–	12.5
6	DWI_ADC	TZ	–	12.5

**Table 3:** Hyperparameters tuned in CNN model. Bold values are considered optimal.

Parameter	Values
Batch size	4, 8, 16, **32**, 64
Training optimization algorithm	**mini-batch SGD**, RMSprop, Adam, Adagrad
Learning rate	0.001, 0.01, **0.05**, 0.1
Momentum	**0.9**
Network weight initialization	random normal, random uniform, **Xavier**
Neuron activation function	**leaky relu**, relu
Weight constraint	0, 0.01, **0.1**, 0.2
Dropout regularization	0, **0.125**, 0.25, 0.5, 0.75

### Statistical analysis and implementation

Our results were interpreted using Python 3.6.9 Jupyter Notebooks (Kluyver et al., 2016), with the SciPy 1.4.1 library (Virtanen et al., 2020) for statistical testing. The two-sided Wilcoxon signed-rank test was employed to analyse the differences between the performance of each model. For learning curve comparisons, tests were performed for each epoch, allowing identification of the epochs for which the model performance differed. We assumed a significance level of *p* < .05. Both models were implemented using Tensorflow 1.12.0 (Abadi et al., 2016), and evaluated on a Windows 10 system with a i7-7700K Intel Core CPU, 32GB RAM, and an NVIDIA GeForce GTX 1080 Ti GPU.

## Results

**Figure 3:**
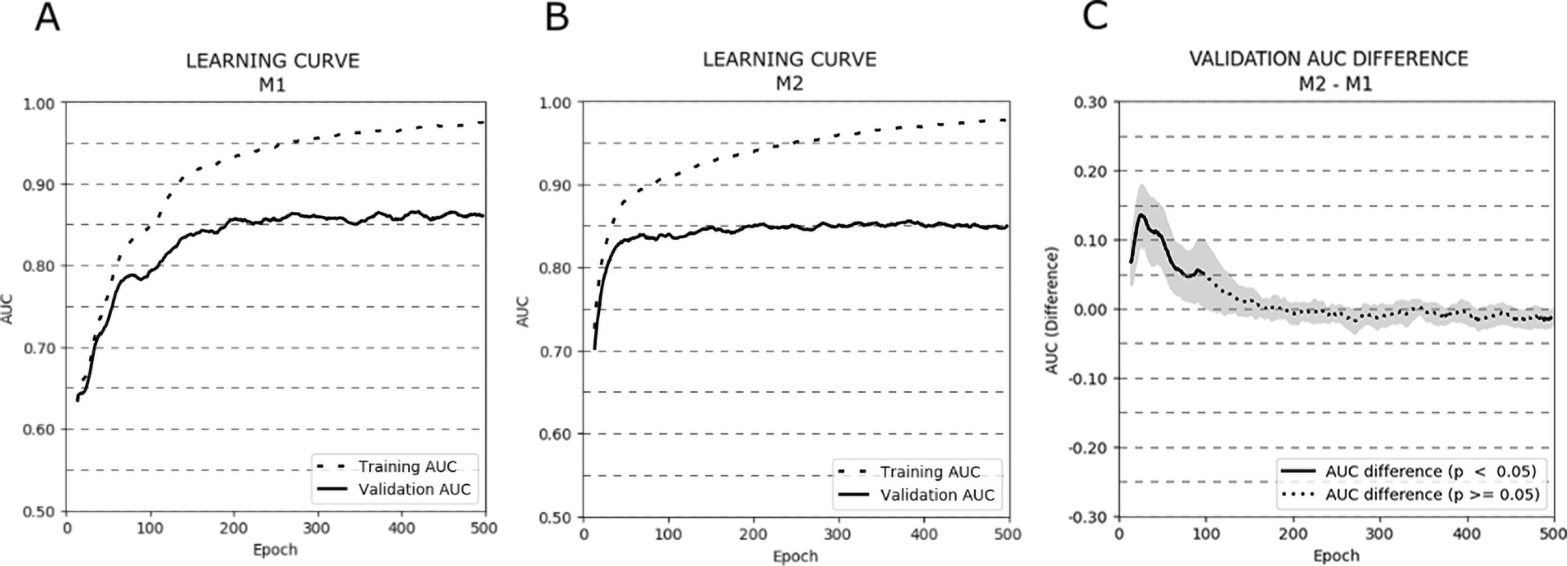
Learning curves (averaged over all cross-validation trials) for training and validation sets for models M1 (A) and M2 (B). Additionally, the AUC difference for the validation set between models M1 and M2 (C) is presented. The bold curves represent epochs with statistically significant differences (*p* < .05). The greyed-out area represents the 95% confidence interval.

Figure 3 depicts the AUC learning curves for models M1 (A) and M2 (B) evaluated on training and validation subsets (cross-validation results were averaged). Additionally, a plot showing the AUC differences between the validation learning curves of both models is presented (C).

The learning curves for both models achieved the same plateau at approximately 0.85 AUC on the validation set. However, model M1 reached that plateau in around 200 epochs, while model M2 approached 0.85 AUC even around 50–75 epochs. The difference in AUC between models M1 and M2 was statistically significant in the first 100 epochs of learning (*p* < .05). To avoid overfitting and to monitor the models’ performance, we repeated the experiment stopping the learning process after 25, 50, 75, and 100 epochs. The mean AUC results for all stopping epochs are presented in Table 4.

**Table 4:** The mean AUC results (averaged for all CV trails, *n* = 10) for both models learned for 25, 50, 75, and 100 epochs.

Model	Mean AUC 25 epochs	Mean AUC 50 epochs	Mean AUC 75 epochs	Mean AUC 100 epochs
M1	0.61	0.72	0.76	0.80
M2	0.76	0.82	0.83	0.84

**Figure 4:**
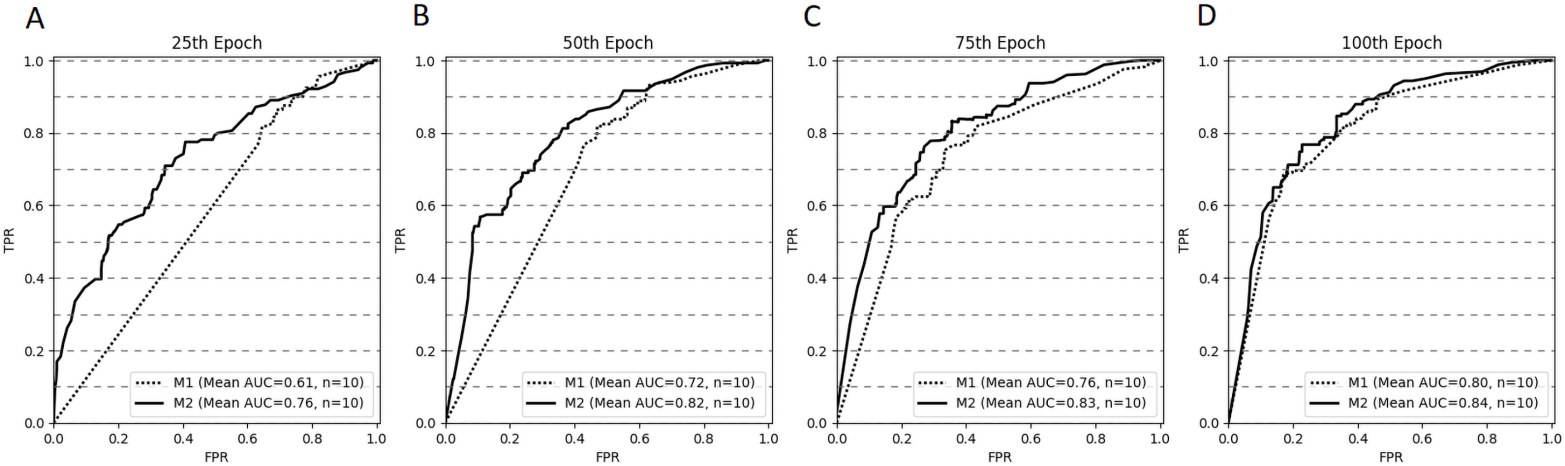
ROC curves (averaged for all CV trails, *n* = 10) for both models stopped after 25 (A), 50 (B), 75 (C) and 100 (D) epochs.

Figure 4 depicts the ROC curves for both models at selected stopping epochs. Comparison of the AUC difference between both models proved to be statistically significant for most of the stopping epochs: the 25th epoch (*AUC*_*diff*_ = 0.15, *Z* = 0, *p* < .001), the 50th epoch (*AUC*_*diff*_ = 0.1, *Z* = 0, *p* < .001), and the 75th epoch (*AUC*_*diff*_ = 0.07, *Z* = 5, *p* < .05). The difference was not statistically significant for the 100th epoch (*AUC*_*diff*_ = 0.04, *Z* = 17, *p* = 0.28).

The optimised models’ AUC results for the validation and test sets are shown in Table 5. The Wilcoxon signed-rank test indicated that the mean of mean CV results (average over all epochs) of model M2 was higher than those scored by model M1 (*Z* = 7, *p* < .05). Model M2 achieved the best AUC (0.84) on the test dataset. We were unable to perform the necessary experiments to check the statistically significant differences of model performance on test set results due to the limitations of the PROSTATEx challenge evaluation platform, which allows only two submissions a day.

**Table 5:** Validation and test set results for models M1 and M2. The mean maximum CV results are comparable with the best results obtained on the PROSTATEx learning dataset.

Model	Mean of mean CV results (AUC)	Mean of maximum CV results (AUC)	Test set result (AUC)
M1	0.831 ± 0.019	0.919 ± 0.016	0.82
M2	0.843 ± 0.021	0.910 ± 0.019	0.84

## Discussion

PI-RADS assessment reduces variability in mpMRI imaging by establishing guidelines, summarising suspicion levels, and standardising reports (Zhang et al., 2018). Additionally, prediction models and risk calculators for prostate cancer can benefit from a combination of the PI-RADS score with risk factors and other clinical features, thus improving their predictive value and optimising clinical diagnostic pathways (Zhang et al., 2018). PI-RADS was proved to have high accuracy for predicting csPCa, and not only radiologists, but also clinical urologists could improve their diagnostic ability by learning the diagnostic process of PI-RADS.

We proposed, designed, trained, and compared two CNN models, both of which supported multi-modal information processing and fusion. In contrast to the M1 model architecture, in which mpMRI series are processed in parallel, and calculated features are simply concatenated to produce a final decision, the architecture of model M2 was optimised to encode domain knowledge, reflecting the PI-RADS diagnostic rules. Analysis of the learning curves reveals that both proposed CNN models suffer from overfitting during learning, although different mitigation techniques have been applied to each, such as data augmentation, regularization, and dropout. Paradoxically, this observation may be associated with strong data augmentation, the necessity of which resulted from the concept adopted of learning models from scratch. The number of training samples exceeded the number of source lesion patterns several times, while artificially generated samples were too alike to each other, causing both models to fit closely to the training set. Data augmentation as a source of overfitting is also confirmed by the stability of the results obtained during model validation, in which additional online data augmentation was not used. Both models achieved a stable plateau, and the AUC scores remain stable as the number of epochs increases.

The most compelling results concern the effectiveness of both models’ learning processes. It can be observed that model M2 converges faster. Validation of the learning curves shows that reaching the 100th epoch can be considered an optimal moment to interrupt learning for model M2; while for model M1, the process should be extended to a minimum of 200 epochs to secure a similar score of 0.85 AUC (the plateau level). It is also noteworthy that model M2, which implements the idea of PI-RADS-inspired prior knowledge encoding in its architecture, scores close to 0.83 AUC after 50 epochs. The AUC difference between the two models is statistically significant, particularly during the first 100 epochs. Validation of the AUC results obtained for both models confirm that model M2 rapidly reaches optimal csPC recognition efficiency, if learned for a limited number of epochs. Model M2 converges faster because it is able to prioritise some solutions (in relation to a lesion’s location in the prostate gland) over others, learning the mutual diagnostic relationships between modalities faster. Model M1 requires more time to discover the same diagnostic relationships between modalities. This demonstrates that diagnostic knowledge is efficiently represented in the network architecture, serving to increase the model’s robustness and stabilise its learning. Faster and more robust learning, as it provides optimal accuracy after a lower number of epochs, can improve the performance of hyperparameter tuning, in which the learning process is repeated many times to discover the hyperparameter combination, that maximises the model’s predictive accuracy.

The faster convergence of model M2, however, does not clearly translate into increased effectiveness, as to a maximum of 500 epochs, both models finally converge to the same AUC value. For the test set, model M2 achieved marginally superior performance, as expressed in its marginally higher AUC value. Despite this, we cannot clearly state that knowledge-based modelling of CNN architecture enables significant improvement.

Certain limitations exist in our research. First, the dataset is of insufficient size to learn from scratch. The resulting problems related to strong data augmentation and overfitting might overshadow the benefits of domain knowledge encoding in CNN model architecture. The use of pre-trained CNN models, or a larger inter-centre dataset could better highlight the advantages of the proposed methodology. Our study would also benefit from comparison with other CNN models that process multi-modal information differently—for instance, by initial fusion of the mpMRI series, in which all information is further processed in a single network, rather than in parallel sub-networks for each modality.

## Conclusions

Encoding domain knowledge in CNN architectures is an important and compelling research subject. The model proposed with domain-knowledge-encoded architecture achieved more stable learning performance and faster convergence to optimal diagnostic accuracy. Although the PI-RADS-inspired model failed to achieve clearly superior results of csPCa classification, those pertaining to the effectiveness of the learning process remain compelling. The results, with some exceptions, also highlight the limitations of PI-RADS-based knowledge-based modelling of CNN model architectures for prostate cancer recognition using mpMRI. These limitations might stem from the limitations of our research, or might indicate that PI-RADS methodology is suboptimal for achieving results that generalise beyond the training data. Encoding domain knowledge in CNN architectures remains a question for researchers. Future studies could explore the application of prior knowledge encoding in the CNN model architectures of other diagnostic applications, in which domain knowledge is also defined in the form of different reporting and data systems, including in breast cancer and BI-RADS, and in lung cancer and Lung-RADS.

## Additional Information and Declarations

### Data Availability

The following information was supplied regarding data availability: Code is available at GitHub: https://github.com/piotrsobecki/PCa-CNNs. Data is available at Cancer Imaging Archive, Collection: SPIE-AAPM-NCI PROSTATEx Challenges: https://wiki.cancerimagingarchive.net/display/Public/SPIE-AAPM-NCI+PROSTATEx+Challenges.
